# Corallivorous Fish Have Reduced Population Sizes and Altered Foraging Behaviour on a Recently Restored Coral Reef

**DOI:** 10.1111/gcb.70590

**Published:** 2025-11-06

**Authors:** Timothy A. C. Lamont, Permas B. Maulana, Ines D. Lange, Muhammad Rizky Madjid, Andi M. A. Pratama, Cicilia V. Parrangan, Tries B. Razak, Nicholas A. J. Graham

**Affiliations:** ^1^ Lancaster Environment Centre Lancaster University Lancaster UK; ^2^ Mars Sustainable Solutions Makassar Indonesia; ^3^ Geography University of Exeter Exeter UK; ^4^ Department of Marine Science and Technology, Faculty of Fisheries and Marine Science IPB University Bogor Indonesia; ^5^ School of Coral Reef Restoration (SCORES), Faculty of Fisheries and Marine Science IPB University Bogor Indonesia; ^6^ General Organization for the Conservation of Coral Reefs and Sea Turtles in Red Sea Jeddah Saudi Arabia

**Keywords:** butterflyfish, coral, coral reef restoration, ecosystem restoration, feeding, fish, movement, selectivity, succession, trophic networks

## Abstract

Ecosystem restoration is a global priority for biodiversity recovery. However, many restoration efforts to date focus only on planting target species, without evaluating the resulting ecosystem‐level impacts on community development and trophic networks. For example, most of the world's efforts to restore tropical coral reefs have evaluated only the recovery of coral organisms. Here, we investigate the re‐establishment of different trophic groups of reef fishes in response to rapid coral recovery at one of the world's largest coral restoration projects. Within 4–6 years of coral restoration starting, coral cover returned to levels found at nearby healthy reference sites. Many groups of fishes recovered similarly quickly; herbivores, planktivores and omnivores recovered abundances equivalent to reference sites within the same time frame. However, although corallivorous fish abundance on 4–6‐year‐old restored reefs was significantly higher than on degraded reefs, it remained at just half the abundance of nearby healthy reference sites. Feeding observations demonstrated that across both healthy and restored habitat, the system's most abundant obligate corallivore (the butterflyfish *Chaetodon octofasciatus* ) consistently targeted a small subset of corals—82% of all recorded bites were on just seven coral morphotaxa. Several of these targeted coral morphotaxa were significantly less abundant on restored reefs than on healthy reference sites. Despite reduced availability of these comparatively rare corals on restored reefs, butterflyfish maintained their dietary preferences, meaning that they exhibited a higher dietary selectivity and foraged over areas twice as large compared to healthy reefs. This demonstrates that despite a rapid recovery of coral cover and some fish groups, the reduced recovery rates of slower‐growing coral morphotaxa limit the speed at which specialist corallivores can re‐establish. Restored coral reefs may regain their coral cover within 5 years, but they will require longer time frames to achieve full trophic networks and ecological complexity.

## Graphical abstract

We investigated the recovery of corals and fishes at one of the world's largest coral reef restoration projects, in central Indonesia. Within 4–6 years of the coral restoration starting, the total amount of coral and many groups of fishes rebounded back to levels equivalent to nearby healthy reefs. However, coral‐eating fish recovered half as fast, because they specialise on a small subset of coral types that were still quite rare on restored reefs. Although coral restoration may generate rapid recovery of some aspects of the ecosystem, restoring the full diversity of corals and fishes will require longer timeframes.

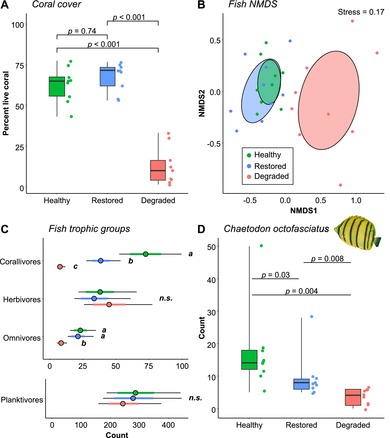

## 1 Introduction

Ecosystem restoration is increasingly recognised as essential to meet global targets aimed at maintaining biodiversity and ecosystem services (Duarte et al. 2020; Strassburg et al. 2020). Defined by the Society of Ecosystem Restoration as “the process of assisting the recovery of an ecosystem that has been degraded, damaged or destroyed”, restoration has an implied target of returning ecosystems to a condition that they would now be in had they not been damaged (Gann et al. 2019). Whilst many ecosystem restoration projects successfully increase the populations of certain target species, the rate at which restored systems deliver a more holistic, ecosystem‐wide recovery is unclear (Carlucci et al. 2020; Harries et al. 2024; Hein et al. 2017; Kollmann et al. 2016; Shimamoto et al. 2018; Warner et al. 2022).

The re‐establishment of species interactions and trophic links in restored ecosystems is central to holistic restoration success (Loch et al. 2020; Parkhurst et al. 2022). Historically, many restoration projects have relied on the ‘Field of dreams’, or ‘Build it and they will come’ hypothesis—the assumption that once an ecosystem's abiotic conditions and dominant vegetation or benthic organisms have been restored, other animals will then naturally return to pre‐disturbance abundances and behaviours (Palmer et al. 1997). However, this assumption is largely untested; in many cases animals do not respond as expected, demanding further understanding of how habitat restoration shapes animal behaviours and populations (Cross et al. 2020; Hale and Swearer 2017). In particular, animals sometimes adjust their feeding strategies in response to habitat restoration; for example, habitat restoration through dam removal caused juvenile salmonids to substantially alter their feeding selectivity and dietary composition in a river in Washington (Morley et al. 2020). Such changes in feeding behaviour could cause knock‐on changes in trophic ecology, population structures and the wider functioning of restored ecosystems (Schmitz et al. 2008; Silliman et al. 2024). Alternatively, in other cases animals are able to maintain the same feeding strategy in both natural and restored habitat; for example, restoration of native plant communities through eradication of invasive macrophytes had no impact on bluegill sunfish feeding behaviour in lakes in Minnesota (Kovalenko et al. 2009).

Coral reefs provide a particularly pertinent example of the need to consider species interactions and trophic ecology in restoration. Reefs are hyperdiverse ecosystems that host many species (Barlow et al. 2018), meaning that attempts to restore corals are likely to have numerous knock‐on impacts on a wide range of other organisms. Despite this, the majority of coral restoration projects worldwide evaluate only changes in coral cover (Boström‐Einarsson et al. 2020; Hein et al. 2017; Razak et al. 2022), so we still lack an understanding of the impact of restoring coral on other reef‐associated animals (Ladd and Shantz 2020; Seraphim et al. 2020; Shaver et al. 2022). Many different functional groups of reef‐associated organisms have the potential to be impacted by coral restoration, because of associated changes to physical reef structure, nutrient cycling and trophic cascades (Seraphim et al. 2020). However, perhaps the most likely group to be directly impacted is the corallivorous butterflyfishes. Many species of butterflyfish are obligate corallivores, whose populations can change rapidly in correlation with hard coral cover (Krimou et al. 2024; Russ and Leahy 2017). These butterflyfishes are often used as model species to understand trophic ecology on coral reefs, with a rich history of studies investigating how their feeding behaviour and dietary specialization are impacted by changes in the wider ecosystem (e.g., Berumen et al. 2005; Cole et al. 2009; Keith et al. 2018; Pratchett et al. 2004; Semmler et al. 2022; Slattery and Gochfeld 2016). As such, corallivorous butterflyfish represent an ideal model system to interrogate the impacts of restoration on trophic linkages in a complex ecosystem.

Here, we use the world's largest coral restoration project as a study system to test whether fish community dynamics recover in parallel with coral regrowth in a restored coral ecosystem. We survey populations of corals and fishes on restored reefs and nearby healthy and degraded reference sites, in order to compare population densities between habitat types. Having identified habitat‐related differences in populations of corallivorous fishes, we then quantify the feeding behaviour of the system's most abundant obligate corallivore, comparing its dietary preferences and foraging movements on healthy and restored reefs. In doing so, we test whether restoring coral is leading to a simultaneous recovery of natural feeding interactions and trophic linkages, or whether recovery of the full fish community lags behind coral regrowth in this restored reef ecosystem.

## 2 Methods

### 2.1 Study System

We carried out this study in May 2023 at the Mars Coral Reef Restoration Project (www.buildingcoral.com) at Pulau Bontosua (Bontosua Island), in the Spermonde Archipelago, South Sulawesi, Central Indonesia, 4°56.9′ S, 119°18.1′ E. This project has been celebrated as a world‐leading case study in regrowing coral at large scales (Saunders et al. 2020). The reef habitat around Pulau Bontosua is made up of a mixture of healthy, degraded and restored areas. Healthy areas have no evidence of historic damage and are generally characterized by live coral cover of > 50%. Degraded areas have been heavily damaged by historic dynamite fishing and coral mining, which primarily occurred 30 years prior to this study (Smith et al. 2021). These degraded areas are generally characterized by live coral cover of < 20%, with the benthos dominated by loose fragments of coral rubble. These rubble fragments are highly motile, and their movement around the benthos is known to preclude the settlement of new corals, preventing natural reef recovery (Ceccarelli et al. 2020; Chong‐Seng et al. 2014; Fox et al. 2003). Restored reefs were historically damaged in the same way as degraded reefs, but underwent active coral restoration for 4–6 years preceding this study. Specifically, the Mars Coral Reef Restoration Project used networks of modular metal frames (called ‘Reef Stars’) to carry out rubble stabilization and coral outplanting on several hectares of reef. Fragments of live coral were attached to Reef Stars and deployed in degraded rubble fields, leading to substantial increases in live coral cover (Williams et al. 2019). Following Reef Star deployment, restored reefs were regularly maintained by manual removal of algae, repairing damage to Reef Stars, and active management of disease outbreaks and algal‐farming damselfish. For full details of the history of the study system and the Mars Coral Reef Restoration Project, see Smith et al. (2021) and Williams et al. (2019).

### 2.2 Coral and Fish Surveys

**FIGURE 1:**
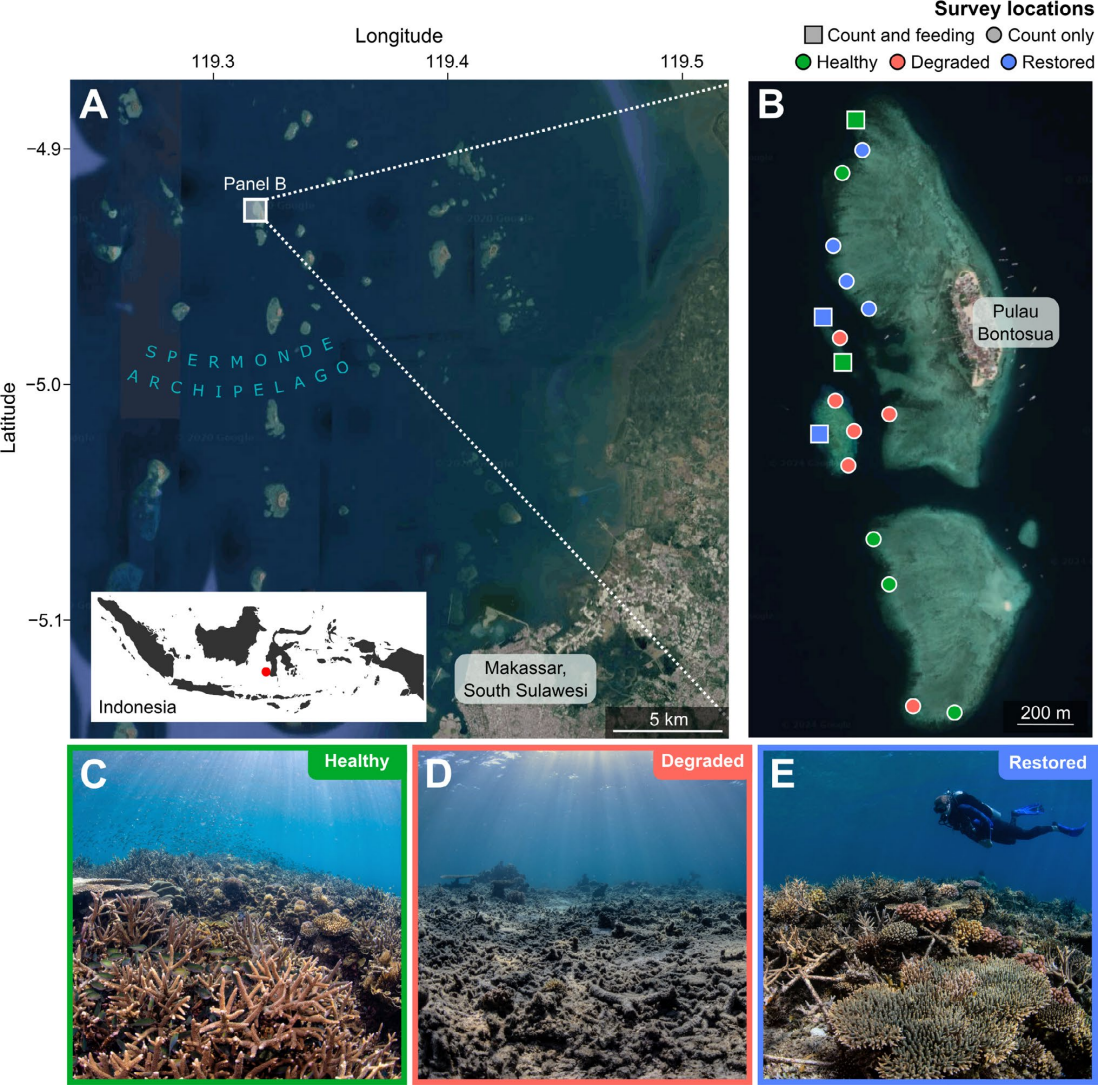
Map of study area. (A) Location of the study system (white box) within the Spermonde Archipelago, and location of the Spermonde Archipelago (red dot) within Indonesia. (B) Location of individual sites for fish and coral counts, and fish feeding observations. (C–E) Representative photos of healthy (C), degraded (D) and restored (E) habitat. Base map satellite images from Google Maps; last accessed on 14/3/2024; map data from Google, CNES/Airbus, Landsat/Copernicus and Maxar Technologies. All photo credits The Ocean Agency.

We carried out coral and fish surveys at six representative sites from each of healthy, degraded and restored reef areas, as part of an annual ecological monitoring programme (Smith et al. 2021). Each of these 18 sites was a 50 × 20 m area, adjacent and running parallel to the reef crest, between 2.0 and 3.5 m depth at low tide. The total tidal range at the study site during surveys was 1.2 m. Each site was at least 50 m from its nearest neighbouring site (Figure 1). At each site, we laid either one or two 50 m transects along the length of the site (two transects per site at half of the sites; one transect per site at the other half of the sites). Where two transects were laid per site, they were laid in parallel, 20 m apart. Different numbers of transects per site were due to time constraints, and each habitat type (healthy, degraded and restored) was described by a total of nine transects.

We measured percentage cover of live coral using benthic photo quadrats. We placed a 0.5 × 0.5 m quadrat at 1 m intervals along each transect, resulting in 51 quadrats per transect. We photographed quadrats from above using a digital camera (Canon G7x Mark III) and analyzed photographs by randomly placing 25 points on each photo using CoralNet software (coralnet.ucsd.edu) and recording the benthic cover beneath each point. We defined benthic cover categories by morphotaxa for live coral, recording both the genus and whether it was a branching or non‐branching growth form. For non‐coral substrate, we specified between ‘other biotic’ (including sponges, macroalgae and turf algae) and ‘abiotic’ (including rock, rubble and sand). We converted these point counts into percentage cover values for each quadrat, and then averaged values across quadrats to generate transect‐level data.

We used the same transects to record fish abundance, using underwater visual census (UVC) on SCUBA. Trained, experienced surveyors in our team counted all fishes within a 5 m belt either side of the transect, identifying them to species level. Surveyors carried out all fish surveys when they first arrived at each site, before any other work (e.g., coral surveys or observations of fish behaviour), to minimize the chance that fish communities were disturbed by the presence of surveyors in the water. We cleaned, checked, managed and stored the data using the Data MERMAID system (datamermaid.org). We excluded cryptic and nocturnal fish species from the survey, and those characterized by (Parravicini et al. 2021) as forming large shoals (more than 50 individuals), because these groups are not possible to count reliably and accurately using UVC. We also excluded fish species that were characterized by (Parravicini et al. 2021) as being mobile within or between reefs, to preclude the possibility of double‐counting fishes with home ranges extending over more than one of our study sites.

### 2.3 Analysis of Coral and Fish Survey Data

We compared the percentage cover of live coral between healthy, degraded and restored habitat using a linear mixed‐effects model, with habitat type as the fixed term and site as a random term. In addition, we compared the percentage cover between habitat types of all coral morphotaxa that comprised more than 5% of the diet of corallivorous fish (see below for quantification of fish diets). There were seven morphotaxa that fulfilled this criteria (see below and Figure S1 for a full list of coral morphotaxa in the diet).

We compared fish communities between healthy, degraded and restored using a non‐metric multidimensional scaling (NMDS) plot to visualize differences in community composition between habitat types. We also split the fish community into four major trophic guilds (corallivores, herbivores, omnivores and planktivores) based on dietary information from (Parravicini et al. 2021) and FishBase (Froese and Pauly 2017). For each trophic guild, we compared fish abundance between habitat types using generalized linear mixed models (GLMMs) with habitat type as the fixed term and site as a random term, choosing the family function (gaussian or negative binomial) based on the best fit to the data in each case. Finally, because corallivores were the only trophic guild to show significant differences in abundance between healthy and restored habitat, we compared the abundance between habitats of *Chaetodon octofasciatus* , the most abundant obligate corallivore at the site. For this comparison, we used non‐parametric pairwise Wilcoxon tests with Benjamini‐Hochberg correction for multiple testing, because extreme outliers meant that no GLMM family functions were able to provide a good fit to these data.

### 2.4 Observations of *C. octofasciatus* Feeding Behaviour

We carried out timed observations of corallivore feeding behaviour at two healthy sites and two restored sites. We did not carry out observations of corallivore behaviour at degraded sites, because corallivore abundance was too low to generate adequate sample sizes in this habitat. We chose *Chaetodon octofasciatus* (the eight‐banded butterflyfish) for observations because it was the most abundant obligate corallivore species in this system. We carried out all observations in the middle of the day (between 9 am and 3 pm), on individual fish that were observed to be actively feeding rather than engaging in courtship or aggressive behaviour.

We selected focal individual fish and followed them on SCUBA. We used several precautions to reduce the chance that our presence would alter the behaviour of the focal fish: we allowed fish to acclimate to our presence by only starting observations after they had resumed feeding in our presence (following (Pratchett et al. 2014)); we maintained a minimum distance of 2 m from the fish at all times (following (MacDonald et al. 2019)); we stayed behind the fish's direction of travel; and we only carried out observations when there were just two observers in the area (no other SCUBA divers or snorkelers nearby). Throughout all observations, there were no signs of the fish being disturbed by our presence (i.e., fishes did not stop feeding, take shelter, or flee from our presence). To avoid double‐counting, for each observation we were careful to choose a fish that was feeding in a different area than we had surveyed before. As *C. octofasciatus* are known to hold aggressively defended feeding territories in monogamous pairs (Ghaffar et al. 2006; MacDonald et al. 2019), this is likely to have reduced the likelihood of double‐counting the same individual, or of counting the partner of a previously observed individual.

We carried out feeding observations on 15 adult fish at each site (30 fish per habitat type) by following focal fish for 3 min, as in (Pratchett 2005). During these focal observations, we estimated the total length of the fish to the nearest cm, and recorded each bite it took of benthic substrate using the same taxonomic classification as in coral population surveys (i.e., identifying corals to morphotaxa). We were careful to differentiate between true bites where the fish's mouth was observed to make contact with benthic substrate, and exploratory investigations where the fish moved its head towards the benthos but did not make contact. When several repeated bites were taken of the same substrate, these were individually counted as separate bites.

### 2.5 Quantification of *C. octofasciatus* Dietary Preferences

First, we compared the total number of bites, and the Shannon diversity of the coral assemblage targeted by all bites, for fishes in healthy and restored habitat. We used a linear mixed‐effects model for both of these comparisons, with habitat type as the fixed term and fish total length and site as random terms.

Following this, we identified coral morphotaxa that comprised more than 5% of the diet of *C. octofasciatus* , which we judged to be the most important dietary targets. Seven morphotaxa fulfilled this criterion; *Galaxea* (20.2% of all bites), other non‐branching coral genera (summed as one group, totaling 17.8%), non‐branching *Porites* (15.2%), *Acropora* (10.7%), *Stylophora* (6.6%), branching *Porites* (5.9%) and non‐branching *Montipora* (5.9%). Together, these seven morphotaxa comprised 82% of all recorded bites. Further, none of the other nine dietary categories recorded (*Hydnophora*, *Isopora*, branching *Montipora*, *Platygyra*, *Pocillopora*, *Seriatopora*, soft corals, solitary corals such as *Fungia*, and other biotic substrates such as sponges and macroalgae) comprised more than 4% of the diet. The relative proportion of all dietary categories is given in Figure S1.

For each of the preferred seven dietary targets, we compared the number of bites taken by fish in healthy and restored habitat, using a generalized linear mixed model (GLMM) with the number of bites taken by each individual fish as the response variable, habitat as a fixed effect, the total number of bites taken by each individual fish (on all substrates) as an offset term, and fish length and site as random terms. We used zero‐inflated GLMMs, choosing the family function (Gaussian or negative binomial) based on best fit to the data in each case.

We then calculated the dietary selectivity for each of the seven most prevalent dietary targets. Following (Graham 2007), we used the Manly selection ratio (Manly et al. 2002), which compares the relative presence of a food resource in the diet with its availability in the environment. We computed selection ratios using the *adehabitatHS* package on R (Calenge and Basille 2023), with a design III approach that matched each individual fish's dietary choices to the availability at the individual site at which it was observed. Values of 1 indicate dietary selection in proportion to environmental availability; greater values indicate positive dietary selection. We tested the effect of habitat on the Manly selection ratio for each dietary item, using zero‐inflated GLMMs with a gamma family function, with habitat type as the fixed term and fish length and site as random terms.

### 2.6 Movement Behaviour

At the same sites, we also carried out timed observations of *C. octofasciatus* movement behaviour during foraging. We chose to carry out observations of movement behaviour independently of feeding behaviour observations (previous section), because we judged that it was not possible for a single observer to accurately record both feeding and movement behaviours at the same time.

We carried out movement observations on 15–16 adult fish at each site (31–32 fish per habitat type), following focal fish for 90 s and estimating their total length to the nearest cm. During these focal observations, we dropped weighted markers at each location of a bite after the fish had left the area, following (Nash et al. 2013). We grouped any repeated bites of the same item together as a single ‘foray’, dropping only a single marker in these cases. After the observation period had ended, we laid down a tape measure that connected all of the foray markers in chronological order. We then recorded the distances between each individual marker as the inter‐foray distance, and the sum of all inter‐foray distances as the total distance travelled per observation time. We also measured the longest axis of the polygon area covered by all markers, and its maximum perpendicular width, as the length and width of the foraging area respectively. We multiplied these values together as a measure of total foraging area, and divided the length by the width as a measure of foraging area compactness (where values of 1 approximate a circular area with maximum compactness, and larger values indicate a more elongated foraging pattern). For each metric, we tested the effect of habitat using GLMMs with habitat as a fixed effect, fish length and site as random terms, and the family function (gaussian, gamma or negative binomial) based on best fit to the data in each case.

### 2.7 Statistical Tools and Packages

We fitted models using the R packages *lme4* (Bates et al. 2015) and *glmmTMB* (Brooks et al. 2017), selecting family functions based on model fit through visual assessment of residual diagnostic plots using the R package *DHARMa* (Hartig 2022) and calculating model estimates using the R package *emmeans* (Lenth 2023). If any random terms were found to explain zero variance, we removed them from the model to avoid overfitting, following (Harrison et al. 2018). We carried out the NMDS analysis using the R package *vegan* (Wagner et al. 2019). We plotted all graphs using the R packages *ggplot2* (Wickham 2016) and *cowplot* (Wilke 2017).

## 3 Results

### 3.1 Coral Cover and Fish Abundance

**FIGURE 2:**
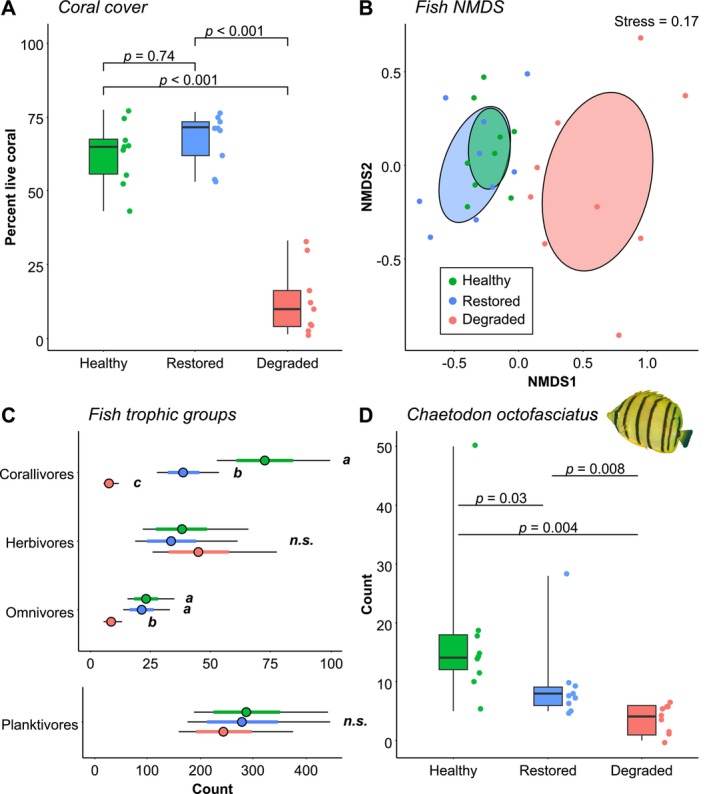
Coral cover and fish abundance on different habitat types. (A) Percentage live coral cover on each habitat type. Each point represents one transect; *p*‐numbers represent pairwise comparisons from GLMMs. (B) NMDS plot of fish communities, coloured by habitat type. Each point represents one transect; ellipses represent standard deviations. (C) Model estimates for abundance of each major trophic group. Points represent mean values; thick coloured error bars represent standard errors; thin black error bars represent 95% confidence intervals. (D) Abundance of *Chaetodon octofasciatus* , the most abundant obligate corallivore in the system. Each point represents one transect; *p*‐numbers represent pairwise Wilcoxon comparisons. Photo of *C. octofasciatus* provided by Rick Stuart‐Smith/Reef Life Survey. In A and D, boxplots represent the median (middle line), interquartile range (boxes) and full range (whiskers) of the data. For full statistical model outputs, see Table S1.

There was a strong effect of habitat type on live coral cover (Linear mixed model (LMM): *F*(2) = 64.1, *p* < 0.001). Both healthy (model estimate ± SE = 62.3% ± 4.05) and restored (66.6% ± 4.05) habitats had approximately five times more coral cover than degraded habitat (10.6% ± 3.81). This difference was significant in both cases (*p* < 0.001), with no significant difference between healthy and restored habitats (*p* = 0.74) (Figure 2A and Table S1).

Overall fish community composition was similar for healthy and restored habitat, which both differed from degraded habitat (Figure 2B). There were no significant differences between healthy and restored habitats in the abundance of herbivores, omnivores or planktivores (Generalised Linear Mixed Models (GLMM): *p* > 0.05 for all; Figure 2C and Table S1). However, healthy habitat (model estimate ± SE = 72.4 ± 11.83) contained nearly twice as many corallivorous fish as restored habitat (38.7 ± 6.47), which in turn contained substantially more corallivores than degraded habitat (7.9 ± 1.56); these differences were all significant (*p* < 0.05 in all cases; Figure 2C and Table S1). This pattern was qualitatively equivalent for the abundance of *Chaetodon octofasciatus* , the most abundant obligate corallivore (Figure 2D). Habitat type significantly affected the abundance of *C. octofasciatus* (Kruskal‐Wallis test: *χ* ^2^(2) = 15.4, *p* < 0.001; Table S1), with healthy habitat supporting significantly higher abundance than restored habitat, which in turn supported significantly higher abundance than degraded habitat (Benjamini‐Hochberg adjusted Wilcoxon paired tests all *p* < 0.05; Table S1).

### 3.2 Availability of Dietary Target Corals in Benthos

**FIGURE 3:**
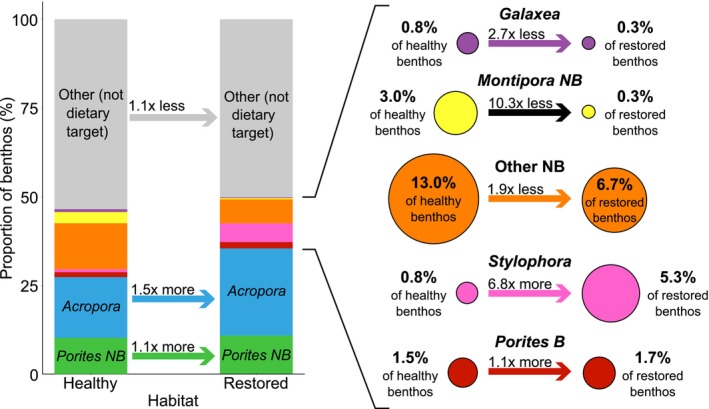
Availability of dietary target corals in healthy and restored habitat. The bar graph shows the proportion of benthic habitat covered by each coral morphotaxa that comprised more than 5% of the diet of *C. octofasciatus* . All other abiotic and biotic habitat are referred to as ‘Other’ (grey bars). Labelled arrows demonstrate the proportional difference between healthy and restored habitat for each group. The inset panel expands the morphotaxa with the lowest proportions in the benthos; the area of each circle is proportional to the availability of that morphotaxon in healthy (left) and restored (right) habitat. B, branching; NB, non‐branching.

The seven coral morphotaxa that each comprised more than 5% of the diet of *C. octofasciatus* (henceforth referred to as dietary target corals) together comprised 46.4% of the benthos in healthy habitat, and 49.9% of the benthos in restored habitat (Figure 3). However, the relative proportions of each dietary target coral in the benthos were considerably different between habitats. Corals with branching morphotypes were generally more prevalent in restored habitat. Specifically, *Stylophora* covered 6.8× more of the benthos in restored habitat compared to healthy habitat, and *Acropora* and branching *Porites* were present in 1.5× and 1.1× higher proportions respectively. By contrast, non‐branching morphotypes were generally less abundant in restored habitat; non‐branching *Montipora* comprised 10.3× less of the benthos in restored habitat, with *Galaxea* comprising 2.7× less and the group of other non‐branching genera comprising 1.9× less—although non‐branching *Porites* comprised 1.1× more of the benthos in restored habitat (Figure 3).

### 3.3 Dietary Composition of *Chaetodon octofasciatus*

**FIGURE 4:**
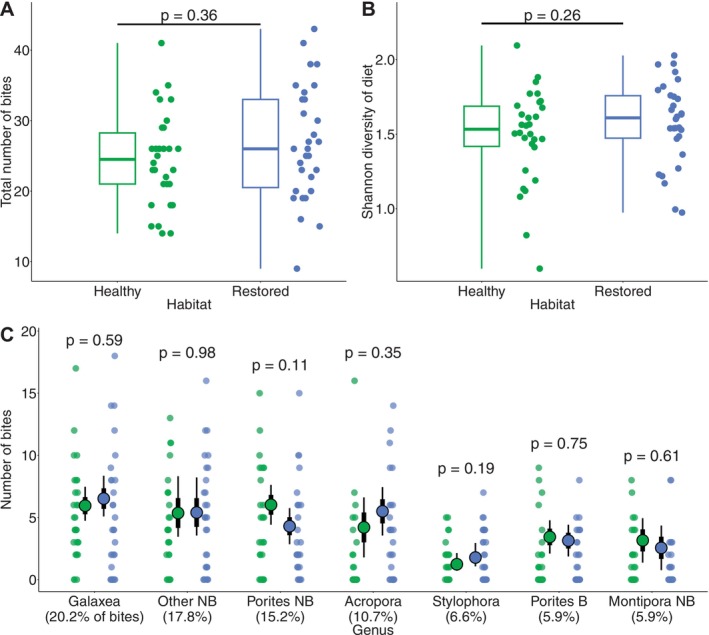
Similar dietary composition of *C. octofasciatus* in healthy and restored habitat. (A) Total number of bites taken in 3 min by each fish; (B) Shannon diversity of all dietary items; (C) Number of bites taken on each of the seven dietary target corals. In each panel, small circular dots represent the diets of individual fish, and *p*‐values indicate statistical comparisons between healthy (green) and restored (blue) habitat. In (A) and (B), boxplots represent the median (middle line), interquartile range (boxes) and full range (whiskers) of the data. In (C), large circles represent the mean estimate from GLMMs; error bars represent standard errors (thick) and 95% confidence intervals (thin). For full details of statistical models, see Table S2. B, branching; NB, non‐branching.

Despite differences in the availability of dietary target corals in the benthos, the dietary composition of *C. octofasciatus* was very similar in healthy and restored habitat. There was no significant difference in the total number of bites taken by individual fish living in each habitat (LMM: *χ* ^2^(1) = 1.38, *p* = 0.36; Figure 4A and Table S2). There was also no significant difference between the Shannon diversity of the diet of fishes living in different habitats (LMM: *χ* ^2^(1) = 1.30, *p* = 0.26; Figure 4B and Table S2), or in the number of bites taken on any of the seven dietary target corals (GLMMs, *χ* ^2^(1) = 0.001–2.58, *p* = 0.11–0.98; Figure 4C and Table S2). This was true even for corals that were present in very low abundance on restored reefs—such as *Galaxea*, which received the highest feeding intensity on restored reefs, despite comprising only 0.3% of the benthos.

### 3.4 Feeding Selectivity by *C. octofasciatus*

In both healthy and restored habitat, there was strong dietary selection [Manly selection ratio > 5] for *Galaxea*, non‐branching *Montipora* and branching *Porites*; moderate dietary selection [Manly selection ratio > 1.5] for non‐branching *Porites* and other non‐branching genera; and no dietary selection [Manly selection ratio ~1; targeted in proportion to its availability in the benthos] for *Acropora*. *Stylophora* was strongly selected for in healthy habitat, but only moderately in restored habitat.

**FIGURE 5:**
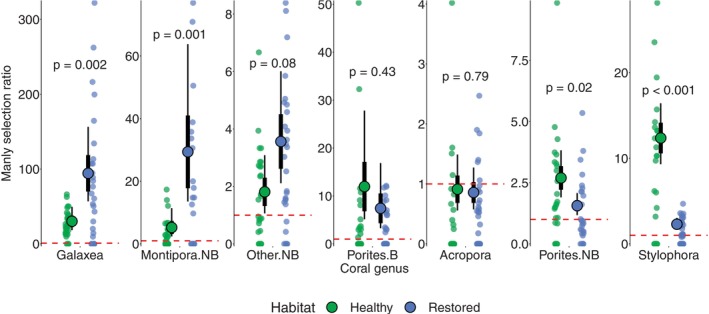
Dietary selectivity (Manly selectivity ratio) of *C. octofasciatus* in healthy and restored habitat. Small dots represent the Manly selectivity ratio of each individual fish; large circles represent the mean estimate from GLMMs; error bars represent standard errors (thick) and 95% confidence intervals (thin); and *p*‐values indicate between‐habitat differences. The dashed red line indicates a value of 1; values above this indicate positive selection for the corresponding genus; values below 1 indicate negative selection. For full details of statistical models, see Table S3. B, branching; NB, non‐branching.

Dietary selection for *Galaxea* and non‐branching *Montipora* was significantly higher in restored habitat than it was in healthy habitat (GLMMs: *χ* ^2^(1) = 9.97–10.2, *p* = 0.001–0.002; Figure 5 and Table S3), because they were targeted with the same number of bites (Figure 4C) despite having lower availability in the benthos (Figure 3). Dietary selection for *Stylophora* and non‐branching was significantly lower in restored habitat (GLMMs: *χ* ^2^(1) = 5.91–97.9, *p* = 0.001–0.02; Figure 5 and Table S3), driven by their comparatively high abundance at restored sites (Figure 3). Selection for *Acropora*, branching *Porites* and the group of other non‐branching corals was not significantly different between habitats (GLMMs: *χ* ^2^(1) = 0.63–3.15, *p* = 0.08–0.79; Figure 5 and Table S3).

### 3.5 Movement Behaviour of *C. octofasciatus*

**FIGURE 6:**
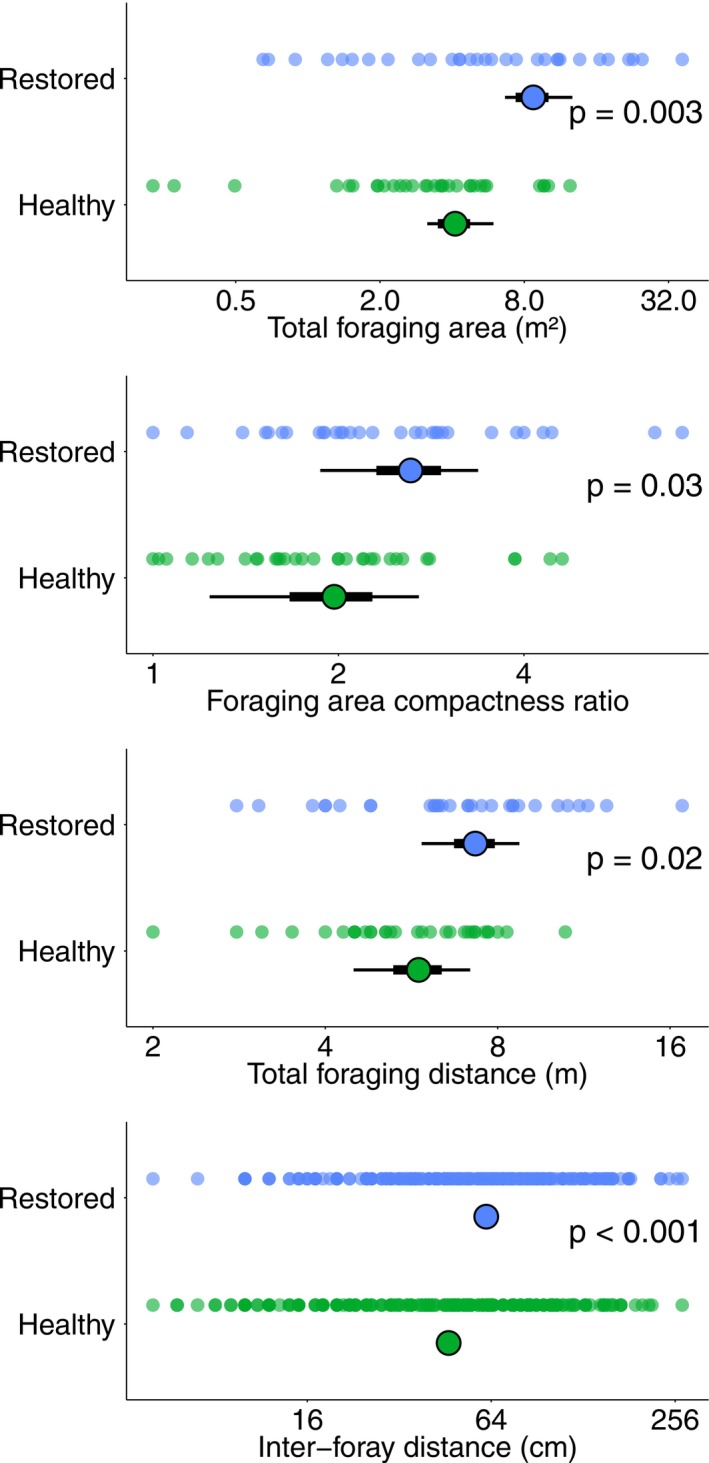
Movement during foraging by *C. octofasciatus* in healthy and restored habitat. Circular points represent the mean estimate from mixed‐effects models; error bars represent standard errors (thick) and 95% confidence intervals (thin); and *p*‐values indicate between‐habitat differences. Note the *x*‐axis scale is log2. For full details of statistical models, see Table S4.

At restored sites, fish foraged over total areas that were twice as large as those at healthy sites (GLMM, *χ* ^2^(1) = 9.58, *p* = 0.003; Figure 6 and Table S4). These larger foraging areas were also significantly more elongated in their shape, with more unidirectional movement patterns leading to increased foraging area compactness ratios (LMM, *χ* ^2^(1) = 4.85, *p* = 0.03; Figure 6 and Table S4). Additionally, fish in restored habitat travelled significantly greater total distances than those in healthy habitats (LMM, *χ* ^2^(1) = 5.50, *p* = 0.02; Figure 6 and Table S4), and exhibited greater inter‐foray distances (GLMM, *χ* ^2^(1) = 24.0, *p* < 0.001; Figure 6 and Table S4).

There was no significant difference in the total length of fishes in each habitat, for either the focal fish in feeding observations (LMM, *χ* ^2^(1) = 0.21, *p* = 0.69) or those in movement observations (LMM, *χ* ^2^(1) = 2.67, *p* = 0.11).

## 4 Discussion

The restored reefs in this study had recovered coral cover and populations of herbivores, omnivores and planktivores that were equivalent to healthy reference sites after 4–6 years. However, this pattern of rapid recovery was not followed by corallivorous fishes. Although populations of corallivorous fishes on restored reefs were significantly higher than on degraded reefs—indicating some recovery—they remained significantly less abundant than on healthy reference sites. This was true both when considering all corallivorous fish species as one group, and when considering only *C. octofasciatus* as the most abundant obligate corallivore. The fact that all other trophic groups had population densities equivalent to healthy reference sites suggests that corallivores were likely to be limited by their diet (which is unique to their group), rather than a need for specific structure or shelter (which is shared by other groups). Indeed, recent work at this site demonstrated that restored and healthy reefs offered equivalent levels of the small‐scale habitat complexity required by small‐bodied reef fish (Vida et al. 2024). Rather, corallivore population sizes appear to be limited by the lower availability of dietary target corals on restored reefs, where three of the seven most prevalent dietary target corals of *C. octofasciatus* had reduced availability in the benthos.

Focal observations of *C. octofasciatus* demonstrated notable consistency in its diet across healthy and restored reefs. Despite reduced availability of dietary target corals in restored habitat relative to healthy habitat, fish living in different habitats exhibited no significant difference in the number of total bites taken per minute, the diversity of targeted corals, or the number of bites on any of the seven most prevalent dietary target corals. This dietary consistency differs from previous studies, which have observed considerable dietary flexibility in *C. octofasciatus* . For example, (MacDonald et al. 2019) assessed the feeding preference of *C. octofasciatus* at different depths in Kimbe Bay (Papua New Guinea), documenting alterations to its diet to match varying resource availability. Other studies have also documented *C. octofasciatus* behaving as a generalist feeder in Redang Island (Malaysia) (Ghaffar et al. 2006) and Thousand Islands (Indonesia) (Madduppa et al. 2014), and able to change its diet to match environmental availability of different corals in Singapore and Malaysia (Feary et al. 2018). However, at this study site, *C. octofasciatus* did not exhibit dietary flexibility, but maintained a consistent dietary preference even when living in habitats with differing food availability. Previous studies have demonstrated that when butterflyfish maintain their preferred diet, they grow faster than when they eat non‐preferred corals (Berumen and Pratchett 2008). In this case, *C. octofasciatus* may be choosing to maintain a diet that is more nutritionally advantageous than switching to corals that are more easily available on restored reefs. Indeed, there was no significant difference in the body size of *C. octofasciatus* between healthy and restored habitat—this suggests that the reduced availability of dietary target corals affected only territory size, rather than causing a decline in body condition.

Several species of the dietary target corals were present in much lower abundance in restored habitat compared to healthy reference sites. Specifically, non‐branching *Montipora* was 10.3 times less abundant, *Galaxea* was 2.7 times less abundant, and other non‐branching genera were 1.9 times less abundant on restored reefs compared to healthy reefs. This reduced abundance of certain non‐branching morphotaxa mirrors similar findings by other studies of these reefs (Lange et al. 2024; Vida et al. 2024). When combined with the consistent dietary preference of *C. octofasciatus* between habitats, this results in significantly higher dietary selectivity for *Galaxea*, non‐branching *Montipora* and other non‐branching genera in restored habitat than in healthy habitat. As a result of this higher dietary selectivity, fish covered twice the foraging area when feeding in restored habitat, with parallel increases in the total distance travelled, inter‐foray distance and compactness ratio (indicating more unidirectional straight‐line swimming). During data collection, we often observed fishes swimming across large patches of non‐dietary target corals (e.g., *Pocillopora*, *Seriatopora*) without feeding, in order to find rare target corals such as *Galaxea* and non‐branching *Montipora*. This observation of increased foraging effort when dietary target corals were less abundant follows classic ecological theory that animals will travel further during foraging when food resources are more scarcely distributed; this has been demonstrated extensively in mammals (Schradin et al. 2010), birds (Marshall and Cooper 2004), fishes (MacDonald et al. 2018), reptiles (Stehle et al. 2017) and invertebrates (Westphal et al. 2006). It is also likely that this increased foraging effort is linked to the reduced population sizes, as increases in territory size mean the reef can support lower population densities (Tricas 1989). Notably, the differences in territory size and population size are equivalent; territory size on restored reefs is twice that of healthy reefs, and population size is half. This correlation indicates that competitive exclusion and increased territory size are likely to be the limiting factors governing butterflyfish abundance on restored reefs. As dietary target corals are prevalent in lower densities, butterflyfish hold larger territories to compensate (whilst maintaining equivalent diets and body sizes), meaning that there are fewer individuals per unit area of reef.

The findings of this study have important implications for restoration design and the expected functional performance of restored reefs over time. Although the restored reefs in this study had developed coral cover equivalent to healthy reference sites, their taxonomic composition of corals was not yet diverse enough to support the same density of corallivorous fishes as healthy reference sites. The abundance of comparatively rare, non‐branching corals was particularly important in this context; for example, *Galaxea* made up less than 1% of the benthos on healthy reefs, but comprised 20% of the diet of *C. octofasciatus* . Although these reefs were originally planted with a range of different corals aiming to approximate natural reference sites (Smith et al. 2021), at this early‐succession stage of their recovery (4–6 years post‐restoration) they have not yet developed an abundance of rare corals equivalent to healthy sites. Although early‐succession restored reefs perform equivalently to healthy systems on some other functions—such as carbonate production (Lange et al. 2024) and small‐scale structural complexity (Vida et al. 2024) – their relative lack of some non‐branching coral genera appears to limit their current carrying capacity for corallivorous fishes. As these restored reefs continue to mature through further successional stages, comparatively rare and slow‐growing corals like *Galaxea* and non‐branching *Montipora* are likely to become more abundant, and the reefs are then likely to support higher densities of corallivorous fish. As such, predictions of restoration outcomes should bear in mind that some measures of ecosystem functioning are not likely to fully recover in the first few years after restoration begins. Rather, the recovery of slower‐growing coral morphotaxa may determine the rate at which it is possible to re‐establish trophic linkages and rebuild the full diversity and functioning of a reef ecosystem.

## Author Contributions

**Timothy A. C. Lamont:** conceptualization, data curation, formal analysis, funding acquisition, investigation, methodology, project administration, resources, software, validation, visualization, writing – original draft, writing – review and editing. **Permas B. Maulana:** data curation, investigation, methodology, project administration, writing – review and editing. **Ines D. Lange:** data curation, investigation, methodology, writing – review and editing. **Muhammad Rizky Madjid:** data curation, investigation, methodology, project administration, writing – review and editing. **Andi M. A. Pratama:** data curation, investigation, methodology, project administration, writing – review and editing. **Cicilia V. Parrangan:** data curation, investigation, methodology, project administration, writing – review and editing. **Tries B. Razak:** conceptualization, funding acquisition, project administration, supervision, writing – review and editing. **Nicholas A. J. Graham:** conceptualization, investigation, methodology, supervision, writing – review and editing.

## Conflicts of Interest

The authors declare no conflicts of interest.

## Supporting information


**Table S1:** Outputs from linear mixed models (LMM), generalized linear mixed models (GLMM), and Kruskal‐Wallis and pairwise Wilcoxon tests (with Benjamini‐Hochberg correction) that investigate the effect of habitat on coral cover and fish abundance (Figure 2 in main manuscript). For mixed‐effects models, overall model statistics are provided in the first row; back‐transformed model estimates and standard errors are provided for the fixed effect (habitat type); and variances ± standard deviations are provided for random terms. For models with an overall significant effect of habitat, the *p*‐values from post hoc Tukey's HSD pairwise comparisons between habitat types are provided.
**Table S2:** Outputs from linear mixed models (LMM) and generalized linear mixed models (GLMM) that investigate the effect of habitat (healthy vs. restored) on number of bites taken by *Chaetodon octofasciatus* on different coral morphotaxa (Figure 4 in main manuscript). Overall model statistics are provided in the first row; back‐transformed model estimates and standard errors are provided for the fixed effect (habitat type); and variances ± standard deviations are provided for random terms. Post hoc Tukey's HSD pairwise comparisons are not provided because only two habitat types were compared (healthy vs. restored).
**Table S3:** Outputs from generalized linear mixed models (GLMM) that investigate the effect of habitat (healthy vs. restored) on Manly selectivity indices by *Chaetodon octofasciatus* on different coral morphotaxa (Figure 5 in main manuscript). Overall model statistics are provided in the first row; back‐transformed model estimates and standard errors are provided for the fixed effect (habitat type); variances and standard deviations are provided for random terms. Post hoc Tukey's HSD pairwise comparisons are not provided because only two habitat types were compared (healthy vs. restored).
**Table S4:** Outputs from linear mixed models (LMM) and generalized linear mixed models (GLMM) that investigate the effect of habitat (healthy vs. restored) on movement behaviour during foraging by *Chaetodon octofasciatus* (Figure 6 in main manuscript). Overall model statistics are provided in the first row; back‐transformed model estimates and standard errors are provided for the fixed effect (habitat type); variances and standard deviations are provided for random terms. Post hoc Tukey's HSD pairwise comparisons are not provided because only two habitat types were compared (healthy vs. restored).
**Figure S1:** Proportion of each coral morphotaxa in the diet of *Chaetodon octofasciatus* , expressed as a percentage of total observed bites. Morphotaxa that comprised more than 5% of total bites (blue bars) were judged to be the most important dietary targets. NB = non‐branching; B = branching.

## Data Availability

The data and code that support the findings of this study are openly available in Zenodo at https://doi.org/10.5281/zenodo.17408240.
