# Supplementary material for: Corallivorous Fish Have Reduced Population Sizes and Altered Foraging Behaviour on a Recently Restored Coral Reef
Source: Glob Chang Biol. 2025 Nov 6;31(11):e70590. doi: 10.1111/gcb.70590 (PMC12590192; doi:10.1111/gcb.70590)
Supplement: Supplementary file 1 — Table S1: Outputs from linear mixed models (LMM), generalized linear mixed models (GLMM), and Kruskal‐Wallis and pairwise Wilcoxon tests (with Benjamini‐Hochberg correction) that investigate the effect of habitat on coral cover and fish abundance (Figure 2 in main manuscript). For mixed‐effects models, overall model statistics are provided in the first row; back‐transformed model estimates and standard errors are provided for the fixed effect (habitat type); and variances ± standard deviations are provided for random terms. For models with an overall significant effect of habitat, the p‐values from post hoc Tukey's HSD pairwise comparisons between habitat types are provided. Table S2: Outputs from linear mixed models (LMM) and generalized linear mixed models (GLMM) that investigate the effect of habitat (healthy vs. restored) on number of bites taken by Chaetodon octofasciatus on different coral morphotaxa (Figure 4 in main manuscript). Overall model statistics are provided in the first row; back‐transformed model estimates and standard errors are provided for the fixed effect (habitat type); and variances ± standard deviations are provided for random terms. Post hoc Tukey's HSD pairwise comparisons are not provided because only two habitat types were compared (healthy vs. restored). Table S3: Outputs from generalized linear mixed models (GLMM) that investigate the effect of habitat (healthy vs. restored) on Manly selectivity indices by Chaetodon octofasciatus on different coral morphotaxa (Figure 5 in main manuscript). Overall model statistics are provided in the first row; back‐transformed model estimates and standard errors are provided for the fixed effect (habitat type); variances and standard deviations are provided for random terms. Post hoc Tukey's HSD pairwise comparisons are not provided because only two habitat types were compared (healthy vs. restored). Table S4: Outputs from linear mixed models (LMM) and generalized linear mixed models (GLMM) that inves [file GCB-31-e70590-s001.docx]

**Supplementary Information for Lamont *et al.*, Corallivorous fish have reduced population sizes and altered foraging behaviour on a recently restored coral reef**

**Table S1.** Outputs from linear mixed models (LMM), generalized linear mixed models (GLMM), and Kruskal-Wallis and pairwise Wilcoxon tests (with Benjamini-Hochberg correction) that investigate the effect of habitat on coral cover and fish abundance (Figure 2 in main manuscript). For mixed-effects models, overall model statistics are provided in the first row; back-transformed model estimates and standard errors are provided for the fixed effect (habitat type); and variances ± standard deviations are provided for random terms. For models with an overall significant effect of habitat, the *p*-values from post-hoc Tukey’s HSD pairwise comparisons between habitat types are provided.

| **Live coral cover** **(panel A)**: LMM, F(2) = 64.1, *p* < 0.001 | |
| --- | --- |
| Healthy habitat (model estimate ± standard error) | 62.3 ± 4.05 |
| Restored habitat (model estimate ± standard error) | 66.6 ± 4.05 |
| Degraded habitat (model estimate ± standard error) | 10.6 ± 3.81 |
| Random effect: site (variance, standard deviation) | 74.16, 8.61 |
| Healthy vs restored comparison *p*-value | 0.740 |
| Healthy vs degraded comparison *p*-value | <0.001 |
| Degraded vs restored comparison *p*-value | <0.001 |
| **Corallivore abundance (panel C)**: GLMM, negative binomial, χ²(2) = 76.3, *p* < 0.001 | |
| Healthy habitat (model estimate ± standard error) | 72.4 ± 11.83 |
| Restored habitat (model estimate ± standard error) | 38.7 ± 6.47 |
| Degraded habitat (model estimate ± standard error) | 7.9 ± 1.56 |
| Random effect: site (variance, standard deviation) | <0.001, <0.001 |
| Healthy vs restored comparison *p*-value | 0.020 |
| Healthy vs degraded comparison *p*-value | <0.001 |
| Degraded vs restored comparison *p*-value | <0.001 |
| **Herbivore abundance (panel C)**: GLMM, Poisson, χ²(2) = 1.99, *p* = 0.37 | |
| Healthy habitat (model estimate ± standard error) | 37.9 ± 10.6 |
| Restored habitat (model estimate ± standard error) | 33.7 ± 10.3 |
| Degraded habitat (model estimate ± standard error) | 45.0 ± 12.5 |
| Random effect: site (variance, standard deviation) | 0.45, 0.67 |
| **Omnivore abundance (panel C)**: GLMM, Poisson, χ²(2) = 12.4, *p* = 0.002 | |
| Healthy habitat (model estimate ± standard error) | 23.2 ± 4.87 |
| Restored habitat (model estimate ± standard error) | 21.4 ± 4.84 |
| Degraded habitat (model estimate ± standard error) | 8.5 ± 1.96 |
| Random effect: site (variance, standard deviation) | 0.23, 0.48 |
| Healthy vs restored comparison *p*-value | 0.960 |
| Healthy vs degraded comparison *p*-value | 0.003 |
| Degraded vs restored comparison *p*-value | 0.012 |
| **Planktivore abundance (panel C)**: GLMM, Poisson, χ²(2) = 0.31, df = 2, *p* = 0.86 | |
| Healthy habitat (model estimate ± standard error) | 287 ± 62.7 |
| Restored habitat (model estimate ± standard error) | 279 ± 66.8 |
| Degraded habitat (model estimate ± standard error) | 245 ± 53.4 |
| Random effect: site (variance, standard deviation) | 0.28, 0.53 |
| ***C. octofasciatus* abundance (panel D)**: Kruskal-Wallis test, χ²(2) = 15.4, *p* < 0.001 | |
| Healthy vs restored Wilcoxon comparison *p*-value | 0.03 |
| Healthy vs degraded Wilcoxon comparison *p*-value | 0.004 |
| Degraded vs restored Wilcoxon comparison *p*-value | 0.008 |

**Table S2.** Outputs from linear mixed models (LMM) and generalized linear mixed models (GLMM) that investigate the effect of habitat (healthy vs restored) on number of bites taken by *Chaetodon octofasciatus* on different coral morphotaxa (Figure 4 in main manuscript). Overall model statistics are provided in the first row; back-transformed model estimates and standard errors are provided for the fixed effect (habitat type); and variances ± standard deviations are provided for random terms. Post-hoc Tukey’s HSD pairwise comparisons are not provided because only two habitat types were compared (healthy vs restored).

| **Total number of bites**: LMM, χ²(1) = 1.38, *p* = 0.36 |  |
| --- | --- |
| Healthy habitat (model estimate ± standard error) | 24.50 ± 1.38 |
| Restored habitat (model estimate ± standard error) | 26.80 ± 1.38 |
| Random effect: site (variance, standard deviation) | 0.152, 0.390 |
| Random effect *fish length* removed due to zero estimated variance. | |
| **Shannon diversity of all bites**: LMM, χ²(1) = 1.30, *p* = 0.26 |  |
| Healthy habitat (model estimate ± standard error) | 1.49 ± 0.06 |
| Restored habitat (model estimate ± standard error) | 1.58 ± 0.06 |
| Random effect: fish length (variance, standard deviation) | <0.001, 0.016 |
| Random effect *site* removed due to zero estimated variance. | |
| ***Galaxea* bites**: GLMM; zero-inflated negative binomial, χ²(1) = 0.29, *p* = 0.59 | |
| Healthy habitat (back-transformed model estimate ± standard error) | 5.95 ± 0.69 |
| Restored habitat (back-transformed model estimate ± standard error) | 6.52 ± 0.83 |
| Random effect: fish length (variance, standard deviation) | <0.001, <0.001 |
| Random effect: site (variance, standard deviation) | <0.001, <0.001 |
| **Other non-branching bites**: GLMM, zero-inflated negative binomial, χ²(1) = 0.001, *p* = 0.98 | |
| Healthy habitat (back-transformed model estimate ± standard error) | 5.36 ± 1.20 |
| Restored habitat (back-transformed model estimate ± standard error) | 5.40 ± 1.16 |
| Random effect: fish length (variance, standard deviation) | <0.001, <0.001 |
| Random effect: site (variance, standard deviation) | 0.061, 0.248 |
| ***Porites* non-branching bites**: GLMM, zero-inflated Gaussian, χ²(1) = 2.58, *p* = 0.11 | |
| Healthy habitat (back-transformed model estimate ± standard error) | 6.02 ± 0.80 |
| Restored habitat (back-transformed model estimate ± standard error) | 4.30 ± 0.72 |
| Random effect: fish length (variance, standard deviation) | <0.001, <0.001 |
| Random effect: site (variance, standard deviation) | <0.001, <0.001 |
| ***Acropora* bites**: GLMM, zero-inflated Gaussian, χ²(1) = 0.91, *p* = 0.35 | |
| Healthy habitat (back-transformed model estimate ± standard error) | 4.20 ± 1.20 |
| Restored habitat (back-transformed model estimate ± standard error) | 5.49 ± 0.97 |
| Random effect: fish length (variance, standard deviation) | 1.736, 1.318 |
| Random effect: site (variance, standard deviation) | <0.001, <0.001 |
| ***Stylophora* bites**: GLMM, negative binomial, χ²(1) = 1.72, *p* = 0.19 | |
| Healthy habitat (back-transformed model estimate ± standard error) | 1.23 ± 0.35 |
| Restored habitat (back-transformed model estimate ± standard error) | 1.78 ± 0.46 |
| Random effect: fish length (variance, standard deviation) | 0.164, 0.405 |
| Random effect: site (variance, standard deviation) | <0.001, <0.001 |
| ***Porites* branching bites**: GLMM, zero-inflated Gaussian, χ²(1) = 0.10, *p* = 0.75 | |
| Healthy habitat (back-transformed model estimate ± standard error) | 3.43 ± 0.67 |
| Restored habitat (back-transformed model estimate ± standard error) | 3.14 ± 0.64 |
| Random effect: fish length (variance, standard deviation) | <0.001, <0.001 |
| Random effect: site (variance, standard deviation) | <0.001, <0.001 |
| ***Montipora* non-branching bites**: GLMM, zero-inflated Gaussian, χ²(1) = 0.26, *p* = 0.61 | |
| Healthy habitat (back-transformed model estimate ± standard error) | 3.15 ± 0.89 |
| Restored habitat (back-transformed model estimate ± standard error) | 2.55 ± 0.89 |
| Random effect: fish length (variance, standard deviation) | 0.780, 0.883 |
| Random effect: site (variance, standard deviation) | 0.805, 0.897 |

**Table S3.** Outputs from generalized linear mixed models (GLMM) that investigate the effect of habitat (healthy vs restored) on Manly selectivity indices by *Chaetodon octofasciatus* on different coral morphotaxa (Figure 5 in main manuscript). Overall model statistics are provided in the first row; back-transformed model estimates and standard errors are provided for the fixed effect (habitat type); variances and standard deviations are provided for random terms. Post-hoc Tukey’s HSD pairwise comparisons are not provided because only two habitat types were compared (healthy vs restored).

| **Selectivity for *Galaxea***: GLMM, zero-inflated Gamma, χ²(1) = 9.97, *p* = 0.002 | |
| --- | --- |
| Healthy habitat (back-transformed model estimate ± standard error) | 30.2 ± 7.61 |
| Restored habitat (back-transformed model estimate ± standard error) | 94.3 ± 24.35 |
| Random effect: fish length (variance, standard deviation) | <0.001, <0.001 |
| Random effect: site (variance, standard deviation) | 0.103, 0.320 |
| **Selectivity for non-branching *Montipora***: GLMM, zero-inflated Gamma, χ²(1) = 10.2, *p* = 0.001 | |
| Healthy habitat (back-transformed model estimate ± standard error) | 5.24 ± 2.08 |
| Restored habitat (back-transformed model estimate ± standard error) | 29.4 ± 11.61 |
| Random effect: fish length (variance, standard deviation) | 0.084, 0.289 |
| Random effect: site (variance, standard deviation) | 0.251, 0.501 |
| **Selectivity for other non-branching genera**: GLMM, zero-inflated Gamma, χ²(1) = 3.15, *p* = 0.08 | |
| Healthy habitat (back-transformed model estimate ± standard error) | 1.81 ± 0.49 |
| Restored habitat (back-transformed model estimate ± standard error) | 3.56 ± 0.95 |
| Random effect: fish length (variance, standard deviation) | <0.001, <0.001 |
| Random effect: site (variance, standard deviation) | 0.113, 0.335 |
| **Selectivity for branching *Porites***: GLMM, zero-inflated Gamma, χ²(1) = 0.63, *p* = 0.43 | |
| Healthy habitat (back-transformed model estimate ± standard error) | 11.96 ± 5.15 |
| Restored habitat (back-transformed model estimate ± standard error) | 7.43 ± 3.11 |
| Random effect: fish length (variance, standard deviation) | <0.001, <0.001 |
| Random effect: site (variance, standard deviation) | 0.294, 0.542 |
| **Selectivity for *Acropora***: GLMM, zero-inflated Gamma, χ²(1) = 0.07, *p* = 0.79 | |
| Healthy habitat (back-transformed model estimate ± standard error) | 0.912 ± 0.229 |
| Restored habitat (back-transformed model estimate ± standard error) | 0.857 ± 0.173 |
| Random effect: fish length (variance, standard deviation) | 0.157, 0.396 |
| Random effect: site (variance, standard deviation) | <0.001, <0.001 |
| **Selectivity for non-branching *Porites***: GLMM, zero-inflated Gamma, χ²(1) = 5.91, *p* = 0.02 | |
| Healthy habitat (back-transformed model estimate ± standard error) | 2.69 ± 0.481 |
| Restored habitat (back-transformed model estimate ± standard error) | 1.56 ± 0.227 |
| Random effect: fish length (variance, standard deviation) | <0.001, <0.001 |
| Random effect: site (variance, standard deviation) | 0.003, 0.051 |
| **Selectivity for *Stylophora***: GLMM, zero-inflated Gamma, χ²(1) = 97.9, *p* < 0.001 | |
| Healthy habitat (back-transformed model estimate ± standard error) | 12.4 ± 1.79 |
| Restored habitat (back-transformed model estimate ± standard error) | 2.3 ± 0.33 |
| Random effect: fish length (variance, standard deviation) | 0.040, 0.201 |
| Random effect: site (variance, standard deviation) | 0.003, 0.058 |

**Table S4.** Outputs from linear mixed models (LMM) and generalized linear mixed models (GLMM) that investigate the effect of habitat (healthy vs restored) on movement behaviour during foraging by *Chaetodon octofasciatus* (Figure 6 in main manuscript). Overall model statistics are provided in the first row; back-transformed model estimates and standard errors are provided for the fixed effect (habitat type); variances and standard deviations are provided for random terms. Post-hoc Tukey’s HSD pairwise comparisons are not provided because only two habitat types were compared (healthy vs restored).

| **Total foraging area (m^2^)**: GLMM, Gamma, χ²(1) = 9.58, *p* = 0.003 | |
| --- | --- |
| Healthy habitat (back-transformed model estimate ± standard error) | 4.12 ± 0.63 |
| Restored habitat (back-transformed model estimate ± standard error) | 8.73 ± 1.36 |
| Random effects *fish length* and *site* both removed due to zero estimated variance. | |
| **Total distance travelled (m)**: LMM, χ²(1) = 5.50, *p* = 0.02 | |
| Healthy habitat (back-transformed model estimate ± standard error) | 5.82 ± 0.57 |
| Restored habitat (back-transformed model estimate ± standard error) | 7.31 ± 0.60 |
| Random effect: fish length (variance, standard deviation) | 0.462, 0.679 |
| Random effect *site* removed due to zero estimated variance. | |
| **Inter-foray distance (cm)**: GLMM, negative binomial, χ²(1) = 24.0, *p* < 0.001 | |
| Healthy habitat (back-transformed model estimate ± standard error) | 46.4 ± 2.01 |
| Restored habitat (back-transformed model estimate ± standard error) | 61.7 ± 2.73 |
| Random effect: fish length (variance, standard deviation) | <0.001, 0.025 |
| Random effect: site (variance, standard deviation) | <0.001, <0.001 |
| **Compactness ratio**: LMM, χ²(1) = 4.85, *p* = 0.03 | |
| Healthy habitat (back-transformed model estimate ± standard error) | 1.97 ± 0.30 |
| Restored habitat (back-transformed model estimate ± standard error) | 2.62 ± 0.32 |
| Random effect: fish length (variance ± standard deviation) | 0.211 ± 0.459 |
| Random effect *site* removed due to zero estimated variance. | |

**Figure S1.** Proportion of each coral morphotaxa in the diet of *Chaetodon octofasciatus*, expressed as a percentage of total observed bites. Morphotaxa that comprised more than 5% of total bites (blue bars) were judged to be the most important dietary targets. NB = non-branching; B = branching.**
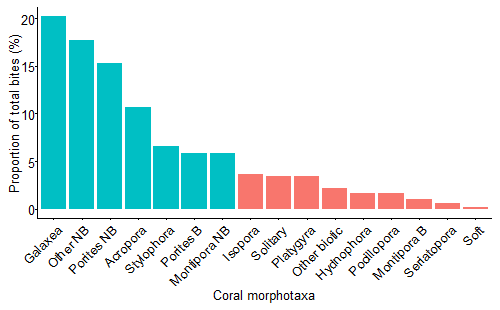
**
